# High-Bandwidth Hysteresis Compensation of Piezoelectric Actuators via Multilayer Feedforward Neural Network Based Inverse Hysteresis Modeling

**DOI:** 10.3390/mi12111325

**Published:** 2021-10-28

**Authors:** Yanding Qin, Yunpeng Zhang, Heng Duan, Jianda Han

**Affiliations:** 1 Tianjin Key Laboratory of Intelligent Robotics, College of Artificial Intelligence, Nankai University, Tianjin 300350, China; qinyd@nankai.edu.cn (Y.Q.); 2120200390@mail.nankai.edu.cn (Y.Z.); 2120180411@mail.nankai.edu.cn (H.D.); 2 Institute of Intelligence Technology and Robotic Systems, Shenzhen Research Institute of Nankai University, Shenzhen 518083, China

**Keywords:** piezoelectric actuator, hysteresis compensation, neural network, inverse modeling, rate-dependent

## Abstract

This paper proposes a feedforward and feedback combined hysteresis compensation method for a piezoelectric actuator (PEA) based on the multi-layer feedforward neural network (MFNN) inverse model. Under the scheme of direct inverse modeling, the MFNN is utilized as the feedforward hysteresis compensator, which can be directly identified from the measurements. The high modeling accuracy and high robustness of the MFNN help to increase the bandwidth of the closed-loop system. Experiments are conducted on a commercial PEA so as to verify the effectiveness of the proposed method. The superimposition of two sinusoidal signals is found to be efficient for the training of the MFNN. Closed-loop trajectory tracking experiments demonstrate that the bandwidth can be increased up to 1000 Hz and the maximum deviation can be maintained closed to the noise level. Meanwhile, there are only two parameters to be tuned in the proposed method, which guarantees ease of use for the inexperienced users. The proposed method successfully realizes high-precision hysteresis compensation performance across a wider frequency range.

## 1. Introduction

The nano positioning system using piezoelectric actuator (PEA) has the advantages of low energy consumption, fast response (millisecond level) and negligible friction [1,2,3,4]. Therefore, it is widely used in various fields, such as precision manufacturing and nano positioning. However, the inherent nonlinearities, such as the hysteresis and creep, will directly affect the performance of the PEA driven positioning system, reduce the control accuracy, and even lead to the instability of the closed-loop system. In order to accurately model and compensate the PEA’s hysteresis, many approaches have been proposed. In general, these approaches fall into the following categories.

Feedforward control: Feedforward control typically involves the hysteresis modeling and inversion. In order to acquire the inverse hysteresis model, the hysteresis model should be obtained first. A lot of models have been used to describe the hysteresis of PEA, such as the Preisach model [5], Krasnosel’skii-Pokrovkii model [6,7], Prandtl–Ishlinskii (PI) model [8], and Duhem model [9]. However, there exist great challenges on how to obtain the accurate inverse model. Although analytical inversion occurs for static PI models, no widely applicable inversion law is available for dynamic (rate-dependent) PI models. In most cases, the exact inversion of the hysteresis model is difficult to construct or even does not exist. To deal with it, the direct inverse modeling (DIM) approach was proposed [10]. However, the performance of feedforward control is highly dependent on the modeling accuracy. In applications, feedforward control can achieve satisfactory hysteresis compensation performance at low frequencies. For high frequency motions, e.g., several hundreds of Hertz, the performance of feedforward control greatly degrades [11,12].

Feedback control: In feedback control, the control signal is generated according to the deviation between the actual and desired trajectories. Therefore, feedback control is capable of suppressing the influence of the uncertainties and external disturbances. The performance of the closed-loop system can be improved [13]. Many controllers were proposed and applied in the hysteresis compensation of PEAs during the past decade [14,15,16,17,18]. In [19], a neural network adaptive control is proposed without constructing the inversion of the hysteresis model. Compared with feedforward control, the frequency range of feedback control can be significantly improved [20]. The frequency range can be expanded to around 200–300 Hz. However, there exist limits for feedback controllers. For even higher frequencies, noise and disturbance induced instability might occur, which should be strictly avoided.

Feedforward and feedback combined control: This combines the advantages of feedforward and feedback controls together where feedforward control is used to model and compensate for the PEA’s hysteresis and feedback control is used to eliminate the modeling error. In [21], a controller is designed for the tracking control of PEAs based on reinforcement learning and inverse compensation. The feedforward and feedback combined control is a candidate to realize high-precision and high-bandwidth hysteresis compensation of PEAs [22]. Based on this framework, many controllers were proposed to improve the control performance [23,24]. However, it is difficult to further increase the bandwidth.

How to achieve high-bandwidth and high-precision hysteresis compensation while reducing the difficulty in tuning of the controller parameters is challenging in the field of hysteresis compensation. Although many effective hysteresis compensation methods have been proposed, they often need to adjust many control parameters onsite. This results in higher requirements on the experience and skills of the users. Unsuccessful tuning of control parameters is inevitable for inexperienced users, which might significantly affect the performance of the overall system. Therefore, the control performances of many hysteresis compensation algorithms are often limited by the skills of the users, resulting in low applicability.

The multilayer feedforward neural network (MFNN) has shown great applicability in modeling nonlinear systems. MFNN can be regarded as a nonlinear composite function. Its learning process is to propagate the input layer by layer along the direction of the network structure to the output layer, and then update the weight and bias through the back-propagation algorithm. The MFNN based hysteresis compensation strategy has been successfully implemented on PEAs to achieve higher bandwidth [21,25,26].

In this paper, a feedforward and feedback combined control is proposed. In the feedforward loop, MFNN is used to directly obtain the PEA’s inverse hysteresis model under the scheme of DIM [10], where the conventional model-inversion process can be avoided. MFNN has been proved to be effective in the hysteresis modeling of PEAs [27]. In this paper, it is used to increase the bandwidth of the overall system with the help of its high modeling accuracy. In the feedback loop, the widely utilized proportional-integral-differential (PID) controller is adopted because of the advantages of simple structure, few parameters, and ease of use.

This paper is organized as follows: Section 2 briefly describes the hardware setup and the characteristics of the inherent hysteresis nonlinearity of the PEA. Section 3 presents the structure of the MFNN and the training process. In Section 4, the systematic control structure was presented. To investigate the efficiency of the proposed method, experimental verifications and performance analyses are provided in Section 5. Section 6 summarizes this paper.

## 2. The Hysteretic Nonlinearity of the Piezoelectric Actuator (PEA)

### 2.1. Experimental Setup

**Figure 1:**
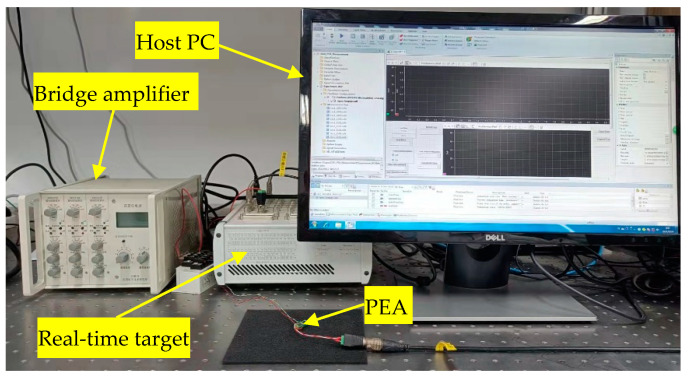
The experimental setup of the system.

As shown in Figure 1, the experimental setup of the system includes a standalone PEA, a high-power piezo driver, a dynamic bridge amplifier for the measurement of the PEA’s strain, i.e., displacement output of the PEA. The closed-loop control algorithm is implemented on a real-time target at a sampling rate of 20 kHz. Detailed information on the experimental setup has been provided in our previous work [13].

### 2.2. Characteristics of the PEA’s Hysteresis

**Figure 2:**
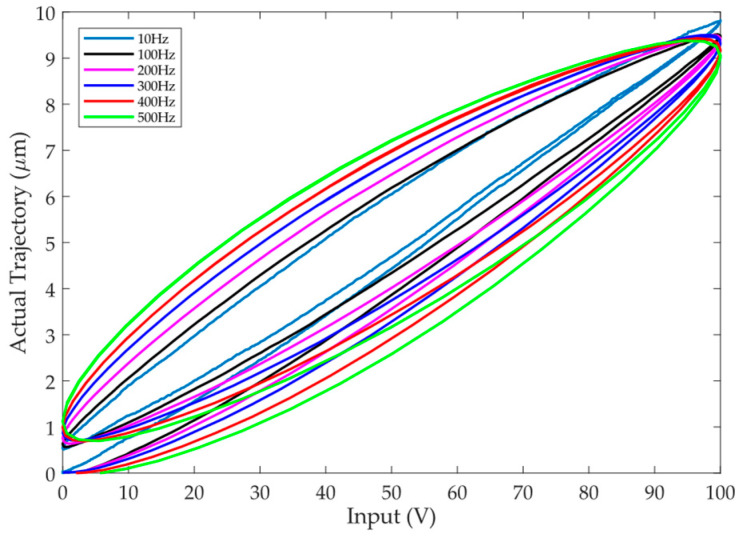
The hysteresis loops of the piezoelectric actuator (PEA) at different frequencies, showing the rate-dependence.

Hysteresis can be approximated as the delay between the input and output of the PEA. For PEAs, hysteresis has become the main factor affecting its positioning accuracy, especially in high-frequency applications. In order to show the hysteresis loops, sinusoidal signals of *u*(*t*) = 50sin(2π*ft* − π/2) + 50 are used to drive the PEA. Take the output displacement as the ordinate and the input voltage as the abscissa, the hysteresis loops of the PEA are shown in Figure 2. The influence of the frequency can be easily observed. The width of the hysteresis loop becomes larger with the increment of the frequency. This is characterized by the so-called rate-dependence. In addition, the hysteresis loops are not strictly symmetrical about the loop center. These characteristics increase the complexity of the system and cause great difficulties in hysteresis modeling and compensation.

## 3. Direct Inverse Hysteresis Modeling Based on Multi-Layer Feedforward Neural Network (MFNN)

**Figure 3:**
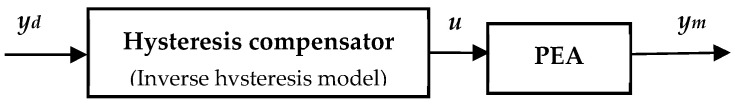
The application of the inverse hysteresis model in feedforward control, where *y_d_* and *y_m_* are the desired and measured trajectories of the PEA and *u* is the control input.

As shown in Figure 3, from the mathematical point of view, the hysteresis of the PEA can be regarded as a nonlinear mapping from the control input *u* to the displacement output of the PEA *y_m_*, i.e., *y_m_* = *f*(*u*). Accordingly, the inverse hysteresis model can be defined as the mapping from the displacement output of the PEA and the control input, i.e., *u* = *f*^−1^(*y_m_*). A straightforward and effective hysteresis compensation strategy is to use the inverse hysteresis model as the hysteresis compensator. As shown in Figure 3, for the hysteresis compensator, the control input is generated using the inverse hysteresis model, i.e., *u* = *f*^−1^(*y*_d_). The control input is then exerted on the PEA. According to the hysteresis of the PEA, the displacement output of the PEA can be expressed as *y_m_* = *f*(*f*^−1^(*y*_d_)) = *y*_d_. If the inverse hysteresis model is accurate enough, the hysteresis of the PEA can be well compensated. As a result, great research efforts have been directed towards the inversion of the hysteresis model. For instance, the PI model has been popular as the inversion of the static PI model can be analytically calculated. Modeling-inversion has become a popular method in recent years. However, this analytical inversion law is not widely applicable for dynamic PI models. Further, the bandwidth of the hysteresis model is limited, i.e., great modeling accuracy can only be achieved within a narrow frequency range. When the frequency increases, the modeling accuracy will drop rapidly. Therefore, how to find an inverse hysteresis model that is generally applicable in a wide frequency range has become a great challenge in the hysteresis compensation of PEAs.

In the meantime, DIM has also been proposed and experimentally tested. It is noted that the inverse hysteresis model can be directly identified using the measured control input and output of the PEA, as shown in *u* = *f*^−1^(*y_m_*). Using DIM, the inversion calculation can be totally eliminated, leading to the increase on the modeling accuracy. Therefore, in this paper, we focus on how to build a high-bandwidth and high-precision inverse model using DIM. The current displacement output of the PEA depends on not only the current input voltage, but also the inputs in previous moments, as investigated in [25]. That means the input–output relationship of the PEA is a many-to-one mapping. Based on this structure, the inverse hysteresis model of the PEA is proposed in this paper so as to increase the bandwidth.

### 3.1. The Structure of the Inverse Hysteresis Model

Based on the research work of Visintin [28], the hysteresis of the PEA depends not only on the input at the present time but also on the operational history of the system. Therefore, in the inverse hysteresis model, the displacements at the current time interval and the previous several time intervals are defined as the model’s input and the physical input to the PEA is defined as the model’s output. If the inverse hysteresis model is cascaded in the feedforward loop, as shown in Figure 3, the hysteresis of the PEA can be compensated. When the modeling accuracy of the inverse hysteresis model is accurate enough, the PEA’s hysteresis can be well compensated.

In this paper, MFNN is adopted to construct the inverse hysteresis model. Basically, the MFNN is a function of system output and input, which can be expressed as follows:

(1) $$u_{FF}(t)=f\left[y_{d}(t),y_{d}(t-1),\cdots ,y_{d}(t-n)\right]$$

where *y_d_*(*t*) and *u_FF_*(*t*) are the desired displacement and the input of the PEA, *n* is the corresponding maximum lags for *y_d_*(*t*), and *f*(·) is a nonlinear mapping reflecting how the historical data affect the current displacement of the PEA.

**Figure 4:**
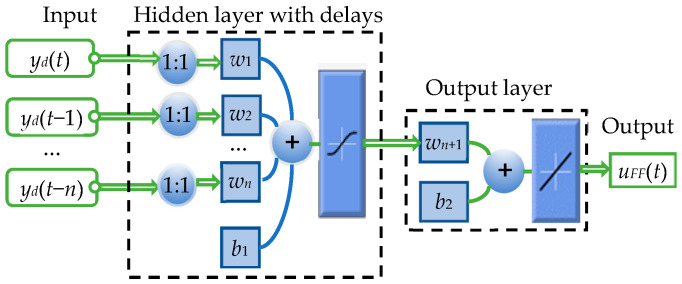
The structure of the multi-layer feedforward neural network (MFNN) for inverse hysteresis modeling.

The schematic diagram of the MFNN can be shown in Figure 4, where *w*_1_, *w*_2_, …, *w_n_* are the weights of the input layer to the hidden layer, *b*_1_ is the hidden layer’s bias, *w_n_*_+1_ is the weight of the hidden layer to the output layer and *b*_2_ is output layer’s bias. The current and previous inputs are utilized to predict the control input with improved modeling accuracy. The activation function of the hidden layer is adopted to be:

(2) $$p(x)=\frac{e^{x}-e^{-x}}{e^{x}+e^{-x}}$$

### 3.2. The Training Process

In this paper, MFNN is adopted as the hysteresis compensator. As a result, the measured input and output of the PEA is utilized in the training of the MFNN. For the training set, it is extremely important to find an appropriate training data so that the modeling accuracy of the MFNN across a wider frequency range can be guaranteed. However, this is very difficult in real implementations. For the type of the input signal in training, the continuous signal can carry more information. In this paper, different training data have been tested, such as the swept sinusoidal signal. It is found that the combination of several basic sinusoidal signal is a very good candidate for the training of the MFNN. The following two sinusoidal signals of *u*_1_(*t*) = 50sin(20π*t* − π/2) + 50 and *u*_2_(*t*) = 50sin(40π*t* − π/2) + 50 are used to drive PEA separately. Subsequently, the measurements are mixed and used as the training set.

The type of the control input and the sampling rate are the two most important factors in the training. A higher sampling rate is necessary in improving the modeling accuracy, whereas the size of the training set will increase significantly, affecting the training time of the network. Furthermore, higher sampling rate leads to higher demands on the equipment and hardware, and high-frequency noise will also be included in the measurement, which might not be an optimal choice. In this paper, a 5 s measurement is used in the training. In order to test the influence of the sampling rate, trainings are implemented using different sampling rate. The results are provided in Table 1. It can be found that a sampling rate of 1 kHz is a better candidate in balancing the training time the modeling accuracy. This facilitates the training of the MFNN. In the control period, however, the sampling rate is set to 20 kHz to guarantee fast response of the closed-loop system.

The lags of the neural network, i.e., the parameter *n* in Equation (1), also affects the modeling accuracy of the MFNN. To determine the maximum lags for *y_d_*(*t*), a set of comparisons have been implemented using different lags in the training of the neural network. The modeling accuracies are given in Table 2. Similar to the hysteresis model, for the inverse hysteresis model, the current control input of the PEA is relevant with its current and historical desired displacements. Therefore, when *n* = 0 (the inverse hysteresis model’s output is only affected by the current desired displacement), the modeling accuracy is low. With the increment of *n*, i.e., *n* = 1, 2, 3, 4, the modeling accuracy can be significantly improved. Although a higher *n* helps to increase the modeling accuracy, the modeling of *n* = 1 to *n* = 4 are comparable. A compromise has to make between the modeling accuracy and the model complexity as higher *n* leads to the remarkable increase on the computational burden and complexity of the neural network. In this paper, *n* = 1 is adopted and the performance of the overall system is further improved using feedback control.

## 4. Feedforward and Feedback Combined Control

**Figure 5:**
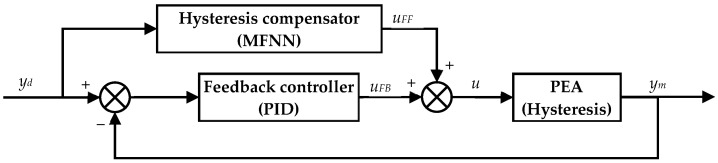
The schematic diagram of the feedforward and feedback combined control.

Due to the inevitable modeling uncertainties of the inverse hysteresis model, it is very difficult for feedforward control to maintain high modeling accuracy across a wide frequency range. In this paper, feedforward and feedback combined control is adopted to further account for the unmodeled dynamics of the system and the external disturbances. In order to reduce the requirements on the user and make the hysteresis compensation ease of use, the architecture shown in Figure 5 is adopted in constructing the feedforward and feedback combined control. The MFNN based inverse hysteresis model previously trained in Section 3 is cascaded in the feedforward loop. Thanks to the high modeling accuracy of the MFNN, the majority of the PEA’s hysteresis can be compensated using the MFNN. The feedback controller is only responsible for the modeling uncertainties and the external disturbances. Therefore, many popular feedback controllers are applicable in the proposed method.

In this paper, the PID controller is adopted as the feedback controller because it is one of the most widely-utilized controllers and the turning of its gains is quite straightforward, thus guaranteeing ease of use. In addition, the integral of the PID controller is very efficient in reducing the steady-state error, making it a very good candidate in eliminating the modeling error of the MFNN.

For the purpose of comparison, another popular feedback controller, i.e., the single-neural adaptive (SNA) controller, is also adopted as the feedback controller. Detailed information on the SNA controller can be found in our previous work [13]. For the SNA controller, the states, the control law, and the parameter updating law are given below:

(3) $$\begin{array}{c} x_{1}(t)=e(t),\;x_{2}(t)=\dot{e}(t),\;x_{3}(t)=\ddot{e}(t),\\ u_{FB}\left(t\right)=u_{FB}\left(t-T\right)+K\cdot \sum_{i=1}^{3}W_{i}(t-T)\cdot x_{i}(t),\\ W_{i}(t)=W_{i}(t-T)+d\cdot e(t)\cdot u_{FB}(t)\cdot x_{i}(t),i=1,2,3 \end{array}$$

where *e*(*t*) is the tracking error, *x_i_*(*t*) is the state of the neuron system, *W_i_*(*t*) is the weight of the state, *K* and *d* are the two parameters that need to be tuned.

## 5. Experimental Verifications

To verify the effectiveness of the proposed MFNN based feedforward and feedback combined control, trajectory tracking experiments are conducted on the PEA. The maximum input voltage is kept below 90 V so as to avoid possible excess of the PEA’s voltage limit. The experiments can be divided into the following three categories:

(1) **Feedforward hysteresis compensation**: the MFNN based inverse hysteresis model is utilized as the hysteresis compensator.
(2) **Feedback hysteresis compensation**: the PID controller is utilized as the feedback controller.
(3) **Feedforward and feedback combined hysteresis compensation**: the proposed method is applied to test the hysteresis compensation performance and the bandwidth of the overall system. In the feedback loop, both the PID and SNA controllers are utilized, and their performances are comparatively analyzed.

For the PEA utilized in this paper, negative voltage is not allowed to be applied so as to avoid the potential damage to the PEA. In this case, the PEA cannot return to its initial position, i.e., the PEA cannot return to origin, as shown in Figure 2. As a result, a bias is added to the reference trajectory, i.e., the reference trajectory does not start from the zero position. This might lead to poor transient behavior for the controllers. In order to obtain a smoother transient performance, the following full-pass filter is applied to the reference trajectory:

(4) $${\ddot{y}}_{d}+2\zeta \omega_{n}{\dot{y}}_{d}+\omega_{n}^{2}y_{d}={\ddot{y}}_{r}+2\zeta \omega_{n}{\dot{y}}_{r}+\omega_{n}^{2}y_{r}$$

where *y_r_* and *y_d_* are the reference and desired trajectories, respectively, the damping ratio is denoted as *ζ*, and the natural frequency is defined as *ω_n_*. The initial conditions are set to be ${\ddot{y}}_{d}={\dot{y}}_{d}=0$. As the transfer function of the filter in (4) is a constant of one, it will not introduce any phase lag. This filter can guarantee that the desired trajectory can always start from the zero initial condition regardless the initial condition of the reference trajectory. After a short period, the desired trajectory will coincide with the reference trajectory. In this paper, *ζ* = 1, and different *ω_n_* is assigned for different reference trajectories. If the frequency is below 100 Hz, *ω_n_* = 200π rad/s, if the frequency is between 100 and 500 Hz, *ω_n_* = 800π rad/s, and if the frequency is between 600 and 1000 Hz, *ω_n_* = 1600π rad/s.

**Figure 6:**
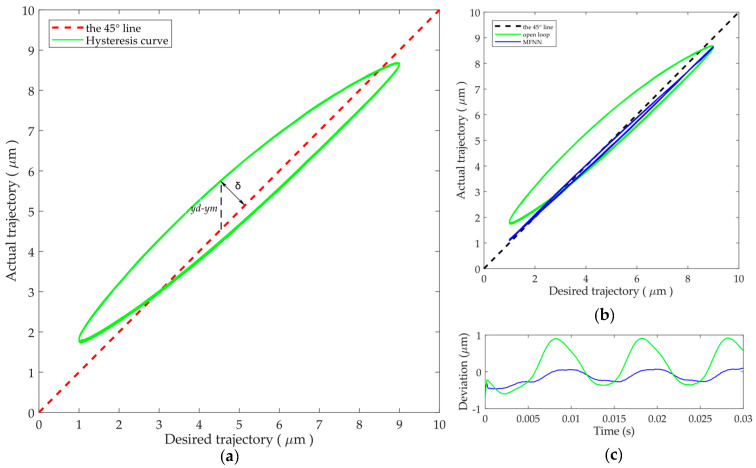
The definition of the deviation used for the evaluation of hysteresis compensation performance: (**a**) the definition; (**b**) the conventional hysteresis plot; and (**c**) the deviation plot.

In order to quantitatively evaluate the hysteresis compensation performance of the controllers, an index shown in Figure 6a is used in this paper, i.e., the distance from each point on the input-output curve of the closed-loop system to the 45° line. This deviation is denoted as *δ* and can be defined as follows [29]:

(5) $$\delta =\frac{y_{m}-y_{d}}{\sqrt{2}}$$

The 45° line in the hysteresis plot means a unitary mapping has been achieved between the desired and actual trajectories. If the hysteresis is totally eliminated, the closed-loop input-output curve should coincide with the 45° line. Poor hysteresis compensation will cause deviations from the 45° line. Essentially, the deviation *δ* measures the width of the hysteresis loop: the smaller the deviation *δ*, the narrower the hysteresis loop. Compared with the conventional hysteresis curve plot, the deviation can better distinguish the differences on the hysteresis compensation performances of difficult controller. This is especially useful when many hysteresis curves need to be compared, as shown in Figure 6b,c. Therefore, the parameter *δ* is utilized to evaluate the hysteresis compensation performance in this paper.

### 5.1. MFNN Based Feedforward Hysteresis Compensation

Feedforward control is employed to verify the effectiveness of the MFNN inverse hysteresis model. The reference trajectory is set to be *y_r_*(*t*) = 4sin(2π*ft* − π/2) + 5, where the frequency *f* is set to be 10, 100, and 200 Hz so as to test the performance across a wide frequency range.

**Figure 7:**
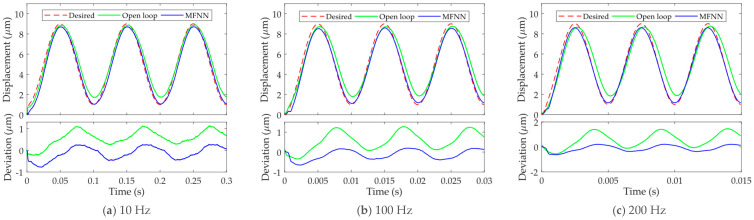
Hysteresis compensation performances of the MFNN based inverse model at different frequencies. (**a**) 10 Hz; (**b**) 100 Hz; (**c**) 200 Hz.

The experimental results are given in Figure 7. Figure 7a shows the tracking of the 10 Hz sinusoidal trajectory, which represents the hysteresis compensation performance of the MFNN at low frequencies. Compared with the open loop results, slight improvement is achieved using the MFNN, whereas the hysteresis is still obvious. Figure 7b,c show the performance of the MFNN at high frequencies, e.g., 100 Hz and 200 Hz. Different from the low-frequency results, it is obvious that the proposed MFNN based inverse hysteresis model achieves high accuracy at higher frequencies. The feedforward trajectories can well follow the desired trajectories. As shown in the deviation plots, the deviation of the compensated system stays close to 0, demonstrating that the hysteresis has been efficiently compensated. Compared with the other feedforward hysteresis compensation, the MFNN based feedforward control can compensate the PEA’s hysteresis across a wider frequency range. This is beneficial to the high-bandwidth hysteresis compensation.

### 5.2. Protional-Integral-Differential (PID) Based Feedback Hysteresis Compensation

**Figure 8:**
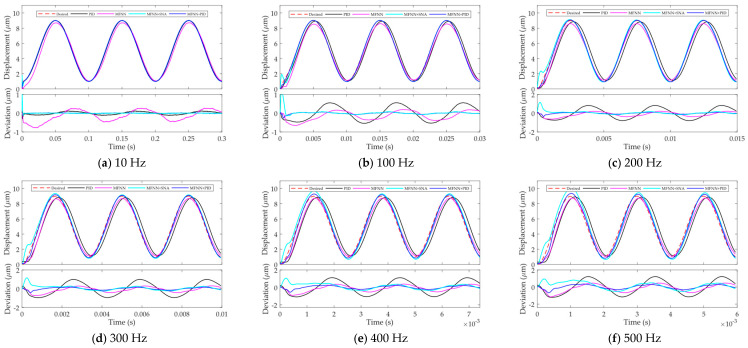
Comparison of the tracking performance across a wide frequency range. (**a**) 10 Hz; (**b**) 100 Hz; (**c**) 200 Hz; (**d**) 300 Hz; (**e**) 400 Hz; (**f**) 500 Hz.

**Figure 9:**
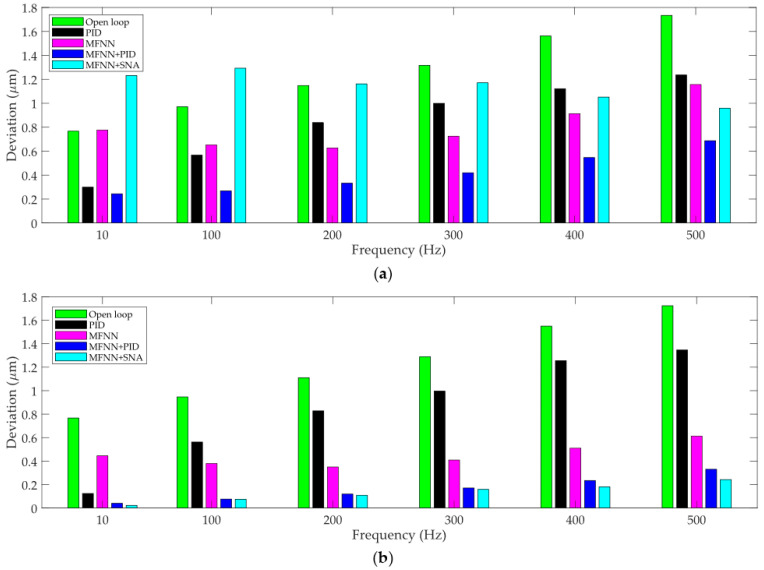
The maximum deviations of the PEA using different controllers: (**a**) in the transient and (**b**) in the steady state.

For the standalone PID controller in pure feedback control, high positioning accuracy can only be achieved when the PEA is required to move to a fixed position. However, its performance in tracking varying trajectory is low. As a result, in trajectory tracking, PID control is frequently used in tracking slow trajectories. For higher-frequency trajectories, the performance of PID control is poor due to the obvious phase lag. For the PID controller, it has been widely adopted that its parameters need to be tuned at different frequencies for a better performance. In this paper, the PID gains are carefully tuned for each trajectory. However, it is very difficult to tune the PID gains for fast trajectories, especially for trajectories higher than 100 Hz. This can be observed in the experimental results in Figure 8 and the statistics on the deviations of the controllers in Figure 9. For slow trajectories, such as the 10 Hz sinusoidal trajectory, the tracking error is comparable with the proposed feedforward and feedback combined control. On the contrary, for frequencies higher than 100 Hz, the tracking error increases obviously.

### 5.3. Feedforward and Feedback Combined Hysteresis Compensation

High-bandwidth hysteresis compensation is pursued in this paper. In this section, tracking of sinusoidal trajectories with even higher frequencies are conducted using the proposed feedforward and feedback combined controller. In order to better compare the performances, both the PID and SNA controllers are adopted as the feedback controllers.

The tuning of the controller parameters is always time-consuming and case-sensitive, affecting its applicability. In order to better test the robustness of the proposed feedforward and feedback combined control, the gains of both the PID and SNA controllers are tuned in tracking low-frequency trajectory. After that, the parameters are fixed throughout the experiments. In this case, the robustness against the rate-dependence and ease of use can be experimentally tested. In this paper, the PID gains are tuned to be *K_p_* = 0.8 and *K_i_* = 3000, and the SNA gains are tuned to be *K* = 0.01 and *d* = 0.4.

The experimental results are shown in Figure 8. Firstly, the tracking performance for the desired trajectories under 200 Hz are analyzed. For the two feedback controllers, the steady-state deviations can be reduced to the noise level, and the steady state performances of the MFNN + PID and MFNN + SNA are comparable. However, in the transient, obvious overshoots can be found in the SNA controller. After a short period, the SNA can follow the desired trajectory well. On the contrary, the standalone PID controller can only maintain good tracking performances at low frequencies. For instance, for the 10 Hz trajectory, the performance of the standalone PID controller is comparable with the proposed method. However, with the increment of the frequency, the performance of the standalone PID controller becomes worse, as shown in Figure 8b–f. As a result, the robustness of the standalone PID control against the rate-dependence is low.

Secondly, high-frequency trajectory tracking introduces huge challenges to the stability and robustness of the controllers. For MFNN + PID and MFNN + SNA, even at high frequencies of 300–500 Hz, the steady-state deviation increases slightly but still stays close to the noise level. In the transient, the obvious overshoot still exists for the MFNN + SNA. On the contrary, for PID control, no matter how to adjust the parameters, it is very difficult to make the PEA follow the desired trajectory for frequencies higher than 200 Hz. This means it is not applicable for high-frequency hysteresis compensation. For the convergence time, the proposed MFNN + PID is fast in response and the system can converge within 1 ms.

The deviations of the system in open-loop, standalone PID control, MFNN based feedforward control, MFNN + PID, and MFNN + SNA are calculated. Without loss of generality, the transient and steady state maximum deviations of the controllers in tracking 10–500 Hz sinusoidal trajectories are shown in Figure 9. It can be seen clearly that with the increase of frequency, the deviations of all the controllers increase. For the 500 Hz desired trajectory, the maximum deviation of MFNN + PID in the transient and steady state can be maintained below 0.69 μm and 0.35 μm, respectively. Similarly, the maximum deviation of MFNN + SNA in the transient and steady state can be maintained below 0.96 μm and 0.24 μm, respectively. This shows that MFNN + PID achieves better transient performance while MFNN + SNA achieves better steady state performance. Regardless of the feedback controller, the proposed MFNN based feedforward and feedback combined control is not very sensitive to the frequency, i.e., high robustness against the rate-dependence is realized. For the other two controllers, the maximum deviation increases obviously at high frequencies.

**Figure 10:**
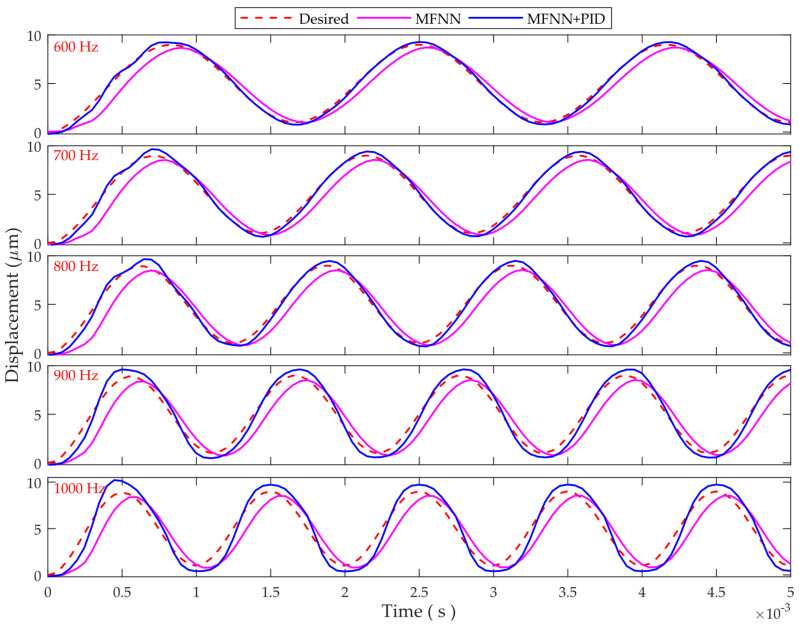
The tracking results of 600–1000 Hz sinusoidal trajectories, showing wide frequency range.

In order to further test the capability of the proposed controller, tracking of 600–1000 Hz sinusoidal trajectories are also implemented. The experimental results are provided in Figure 10. For the standalone PID control, instability problem occurs, and for the MFNN + SNA, the tracking performance is poor. Therefore, only the tracking results of MFNN and MFNN + PID are presented. It can be observed that MFNN + PID can track the desired trajectory well for 600–800 Hz trajectories. For the 900 and 1000 Hz trajectories, the tracking performance degrades, and small deviations can be found between the measured and desired trajectories. However, MFNN + PID is still effective in hysteresis compensation for such high-frequency trajectories.

Based on the above experimental results, high-bandwidth and high-precision hysteresis compensation has been achieved using the proposed MFNN based feedforward and feedback combined control. The trajectory tracking performance has been significantly improved for frequencies up to 1000 Hz. This is beneficial to the fast positioning and manipulation tasks.

It must be noted that the PID controller might not be the best feedback controller in the proposed feedforward and feedback combined control. As shown in experimental results in Figure 8 and Figure 9, the steady state performance of the SNA controller is superior to the PID controller because a slight decrease on the maximum deviations can be observed in the combination of MFNN + SNA. However, the combination of MFNN + PID is still recommended in that PID is widely-used and easy to implement. The tuning of the PID gains is also quite straightforward.

## 6. Conclusions

In this paper, the MFNN based feedforward and feedback combined control is proposed to compensate the PEA’s hysteresis. The MFNN is used construct the feedforward hysteresis compensator under the scheme of direct inverse modeling. In the feedback loop, the widely utilized PID controller is adopted. Experimental verifications show that high-bandwidth modeling accuracy has been achieved by the use of the MFNN as the inverse hysteresis model. Meanwhile, the introduction of MFNN successfully avoids the complex inversion problem of the conventional model-inversion approaches. The experimental results of tracking sinusoidal trajectories with frequencies up to 1000 Hz show that the proposed method can successfully compensate the rate-dependent hysteresis of the PEA. In addition, there are only two parameters need to be tuned in the proposed method. They can be easily tuned at a low frequency and fixed throughout the experiments. This greatly reduces the requirements on the experience and skills on the operator, thus guaranteeing ease of use.

## Figures and Tables

**Table 1:** The modeling accuracy of the proposed multi-layer feedforward neural network (MFNN) model using different *n*.

Sampling Rate (Hz)	Training Time (s)	MS Error (V^2^)	Regression R Values
100	8	0.68	0.957
1 k	114	0.001	0.999
10 k	917	0.015	0.999

**Table 2:** The modeling accuracy of the proposed MFNN model using different *n* values.

*n*	MS Error (V^2^)	MAX Errors (V)	Regression R Values
0	0.5075	1.465	0.980
1	0.0013	0.7206	0.999
2	0.0010	0.6191	0.999
3	0.0008	0.5132	0.999
4	0.0007	0.4453	0.999
